# The therapeutic use of music for chronic pain: a psychological and neurobiological perspective

**DOI:** 10.3389/fpain.2025.1658523

**Published:** 2026-01-13

**Authors:** Víctor Fernández-Dueñas, Antoni Rodríguez-Fornells, Jennifer Grau-Sánchez

**Affiliations:** 1 Pharmacology Unit, Department of Pathology and Experimental Therapeutics, School of Medicine and Health Sciences, Institute of Neurosciences, Universitat de Barcelona, Barcelona, Spain; 2 Neuropharmacology & Pain Group, Neuroscience Program, IDIBELL-Bellvitge Institute for Biomedical Research, Barcelona, Spain; 3 Cognition and Brain Plasticity Unit, Department of Cognition, Development and Educational Psychology, Faculty of Psychology, University of Barcelona and Bellvitge Institute for Biomedical Research, Barcelona, Spain; 4 Catalan Institution for Research and Advanced Studies (ICREA), Barcelona, Spain; 5 Research Group on Complex Health Diagnoses and Interventions from Occupation and Care (OCCARE), EUIT University Center, Autonomous University of Barcelona, Terrassa, Spain

**Keywords:** music, chronic pain, analgesia, multimodal therapy, side-effects

## 1 Introduction

Chronic pain, defined as pain lasting more than three months, is a leading cause of disability and a major reason for seeking medical care. Prevalence estimates vary widely, with research indicating that between 10% and over 50% of the adult population is affected, with higher rates observed among women, socially vulnerable groups, and older adults (1). Although symptoms and experiences differ greatly from person to person, chronic pain negatively impacts physical, mental, emotional, and social well-being, and often coexists with mood disorders such as depression and anxiety. In addition, chronic pain may increase the risk of mental health issues, suicide, and substance misuse (2).

In recent years, there has been growing recognition that chronic pain is not merely a biological phenomenon, but a complex, multidimensional condition shaped by psychological, social, and cultural factors (1). This understanding has led to the development of multimodal and interdisciplinary approaches to pain management, which aim to address the full spectrum of the pain experience. Sole reliance on pharmacological treatments fails to fully address this complexity and carries significant risks, including tolerance, dependence, and adverse effects (3). Among the complementary strategies being explored, music-based interventions have gained increasing attention due to their accessibility, safety, and potential to modulate neurochemical, physiological, and psychological processes (4–8).

Of note, recent meta-analyses have emphasized the importance of distinguishing between passive music listening, typically administered without therapeutic guidance, and music therapy, which includes active engagement with music facilitated by a trained therapist. Evidence suggests that the latter may yield more consistent and clinically significant outcomes, particularly in complex conditions such as chronic pain (6, 7, 9). However, the selection of a specific type of intervention, whether passive music listening or active music therapy, should be guided by clinical context, patient preferences, therapeutic goals, and available resources. For instance, in long-term settings, patient acceptance is high, as, compared to other treatments, music listening is frequently regarded as more enjoyable and carries less social stigma (10). Thus, music listening would be particularly well-suited to person-centered care models that emphasize emotional well-being, autonomy, and active patient engagement.

In this opinion article, we aim to contribute to this interdisciplinary dialogue by offering a neurobiologically grounded perspective on the therapeutic potential of music in chronic pain management. Drawing on our background in neurobiology and cognitive neuroscience, we focus particularly on the role of the dopaminergic system and its disruption in chronic pain, proposing that music may act not only as a pleasant distraction but also as a means to restore reward-related brain function. By articulating the mechanisms through which music may exert analgesic effects, we hope to support the integration of music-based interventions into broader, evidence-informed models of care.

## 2 Music-based interventions in chronic pain

Although randomized controlled trials specifically examining the effects of music on chronic pain remain relatively limited [e.g., (11–13)], a growing body of experimental and clinical research suggests that music-based interventions can reduce pain perception and alleviate associated symptoms such as anxiety and depression (14–16). Meta-analyses and systematic reviews have reported modest-to-moderate reductions in pain intensity, along with improvements in mood and quality of life across a range of chronic pain conditions, including fibromyalgia, osteoarthritis, neuropathic pain, and cancer-related pain (6, 8).

The beneficial effects of music in pain relief likely arise from a synergistic and dynamic interplay of psychological, physiological, and neurobiological mechanisms. Engaging in musical activities, whether listening, singing, or playing, has the unique ability to evoke pleasurable feelings and a wide range of emotions, playing a particularly important role in mood regulation (17). In the context of chronic pain, music can elicit positive emotional states that counteract the negative affect often associated with persistent pain. Additionally, it promotes relaxation and reduces stress responses, contributing to parasympathetic activation and lowering physiological arousal. These effects, such as reduced heart rate, blood pressure, and muscle tension, can further diminish pain perception (18).

Music also engages perceptual and cognitive functions, capturing attention and redirecting focus away from pain. This attentional redirection is especially effective when the music is emotionally engaging, familiar, or personally meaningful, as it competes with nociceptive input for cognitive resources. These psychological processes may influence cognitive appraisal, enabling individuals to reframe pain as less intrusive or threatening (17). Together, these mechanisms enhance coping strategies and contribute to music's overall therapeutic impact. Of note, theoretical models such as the neuromatrix (19) have emphasized the role of central processing, attention, and emotion in shaping the pain experience. These models support the idea that music may influence pain not only through distraction but also by modulating affective and cognitive dimensions.

## 3 Neurobiological mechanisms

From a neurobiological point of view, extensive research suggests that music may exert its effects by engaging multiple pathways that modulate pain perception (20, 21). Recent reviews have outlined in detail the neurobiological pathways through which music may produce analgesic effects, including the involvement of β-endorphins, oxytocin, and dopamine, and their interactions across central and peripheral systems (4, 14, 20). While these accounts span a broad spectrum of mechanisms, our perspective focuses specifically on the dopaminergic system and its disruption in chronic pain, which we consider a particularly promising target for music-based interventions.

Neuroimaging studies using PET and fMRI have consistently shown that listening to pleasurable music triggers dopamine release in the nucleus accumbens (NAc) and activates the ventral tegmental area (VTA), key nodes of the mesolimbic reward system (22–26). Interestingly, this dopaminergic response occurs both during the anticipation of musical climaxes and at the peak of emotional experience, with distinct anatomical patterns: the caudate is more involved in anticipatory phases, whereas the NAc predominates during peak pleasure signaling (25). Similarly, pharmacological studies provide causal support for dopamine's role in music-induced reward. For instance, enhancing dopaminergic transmission with levodopa increases hedonic and motivational responses to music, while blocking dopamine receptors with risperidone reduces them signaling (23). This demonstrates that dopamine is not merely associated with musical enjoyment but is a necessary mediator of its rewarding properties.

Beyond these subcortical hubs, music also engages cortical regions involved in reward valuation, affective regulation and decision-making, including the orbitofrontal cortex (OFC), ventromedial prefrontal cortex (vmPFC), anterior cingulate cortex (ACC), and insula (20, 26). These areas do not operate in isolation. They form a dynamic network that integrates sensory input from auditory cortices with limbic signals, shaping the subjective experience of pleasure and its motivational significance. The OFC and vmPFC, for instance, contribute to assigning value and predicting reward, while the ACC and insula mediate affective salience and interoceptive awareness, functions that are critical in both pain perception and emotional regulation (20, 27). This distributed architecture underscores that music-induced pleasure is an emergent property of coordinated activity across reward, affective, and cognitive circuits, rather than a localized phenomenon. Dopaminergic signaling plays a central modulatory role in this integration, facilitating communication between subcortical reward hubs and prefrontal regions involved in valuation and control (28).

Chronic pain is consistently associated with structural and functional alterations in brain networks that mediate reward, motivation, and affective regulation. Neuroimaging studies reveal disrupted connectivity between the mesolimbic system, particularly the nucleus accumbens (NAc) and ventral tegmental area (VTA), and prefrontal regions such as the mPFC and ACC, which are critical for valuation and top-down control. These changes are accompanied by reduced dopaminergic signaling and gray matter loss in reward-related hubs, contributing to symptoms such as anhedonia, apathy, and diminished motivational drive (29–33). These alterations are thought to impair the brain's capacity to generate and sustain positive affective states, which may exacerbate pain chronification and comorbid depression. Targeting these disrupted dopaminergic circuits through interventions such as music may therefore represent a promising strategy to restore reward processing and motivational drive.

The convergence of neuroimaging, behavioral, and pharmacological evidence provides a compelling rationale for investigating dopaminergic pathways as central mediators of music-induced analgesia. Nevertheless, the current evidence base is constrained by small sample sizes and methodological heterogeneity, which limits the generalizability of findings and underscores the need for more rigorous designs. In addition, beyond dopaminergic signaling, other neurochemical systems, particularly the endogenous opioid system, also appear to contribute to music-induced analgesia. Music may reduce opioid receptor availability or enhance dopaminergic activity through opioid-dopamine interactions (34, 35). However, recent studies using opioid antagonists indicate that opioid blockade primarily influences physiological arousal rather than subjective pleasure (36, 37). This physiological arousal may potentiate secondary mechanisms, such as attentional distraction, relaxation, and positive affect (38).

All these effects provide a strong rationale for incorporating music as an adjuvant to analgesic drugs, particularly opioids, offering both physical and emotional relief while mitigating risks associated with high-dose pharmacotherapy. However, individual variability in musical responsiveness may play a critical role in determining therapeutic outcomes. The perceived pleasantness of music is essential for analgesic effects, underscoring the need to tailor interventions to personal preferences and motivational profiles (39, 40). Patient engagement, shaped by beliefs, values, and prior experiences, further influences adherence and efficacy. In addition, commitment to consistent engagement and support from family, friends, and healthcare providers further increases adherence and effectiveness of these interventions in managing chronic pain (41, 42). Advancing our understanding of these mechanisms and moderators will be crucial for developing targeted, evidence-based applications of music in clinical pain management.

## 4 Discussion

The growing interest in music-based interventions for chronic pain reflects a broader shift in pain science toward integrative, patient-centered approaches. While pharmacological treatments remain central in clinical practice, their limitations, particularly in long-term use, have prompted the exploration of complementary strategies that address the emotional, cognitive, and social dimensions of pain. Music, as a complex auditory stimulus capable of eliciting strong affective and motivational responses, offers a unique therapeutic possibility. Its ability to engage reward circuits, modulate attention, and influence emotional regulation suggests that it may act on multiple levels of the pain experience simultaneously. To fully understand this therapeutic potential, it is essential to examine the neurobiological mechanisms underlying music's effects on pain. Among these, dopaminergic pathways have emerged as particularly relevant because of their central role in reward processing and motivation, functions often disrupted in chronic pain. By emphasizing this mechanism, we aim to provide a clearer neurobiological framework that strengthens clinical relevance and guides the development of more targeted interventions.

The therapeutic relevance of dopaminergic pathways in chronic pain is supported by converging evidence from neuroimaging, pharmacological, and behavioral studies. Functional imaging research indicates that chronic pain patients exhibit hyporesponsivity to natural rewards and impaired prediction error signaling, consistent with a reward deficiency model of pain chronification (4, 20). These deficits exacerbate negative affect and diminish engagement with adaptive behaviors, creating a vicious cycle that sustains pain and emotional distress. By re-engaging these circuits through music, dopaminergic modulation could help restore adaptive network dynamics, offering a mechanistic pathway for alleviating both sensory and emotional dimensions of chronic pain. Music's ability to activate mesolimbic and prefrontal regions involved in reward valuation suggests that it may counteract the hypodopaminergic state characteristic of chronic pain, thereby improving motivation and affective flexibility. Different types of music interventions may engage these systems to varying degrees: active music-making may more strongly recruit motor and reward circuits, whereas passive listening may primarily modulate affective and attentional processes (20, 43). Understanding these distinctions is essential for tailoring interventions to individual needs and clinical contexts.

Recent findings also highlight the potential of music to restore disrupted neural connectivity in chronic pain conditions. Functional imaging studies have shown that music-based interventions can enhance activity in regions implicated in both pain modulation and emotional processing, such as the ACC, insula, and PFC (20, 44, 45). These effects may counteract maladaptive neuroplastic changes associated with chronic pain, including dysregulation of the mesolimbic reward system. Music's ability to influence supraspinal regions involved in both pain and emotion suggests a shared neurobiological substrate (4). Importantly, music's capacity to evoke positive affect and foster a sense of agency may be particularly valuable in populations where chronic pain coexists with depression or anxiety.

Despite these promising insights, the translation of music-based interventions into routine clinical practice remains limited, partly due to variability in study designs and a lack of consensus on optimal implementation strategies. Increasing sample sizes is often recommended, but this can be challenging in the context of personalized interventions and heterogeneous pain experiences. Alternative strategies to strengthen the evidence base include the use of within-subject designs, which can control for individual variability, and longitudinal studies that assess sustained effects over time. A recent review of over 120 clinical trials identified intervention duration as one of the strongest predictors of effectiveness in music therapy for chronic pain, emphasizing the need to consider not only the type of intervention but also its intensity and continuity over time (8). Combining self-reported outcomes with neuroimaging and psychophysiological measures may provide more objective insights into underlying mechanisms and treatment efficacy.

Future research should aim to consolidate the evidence base through methodologically rigorous trials that account for individual variability in musical responsiveness and preferences. It will be essential to move beyond short-term interventions and examine sustained effects in real-world clinical settings where adherence and ecological validity are critical. Investigating how music interacts with other therapeutic modalities, such as cognitive-behavioral therapy, mindfulness, or physical rehabilitation, could also yield valuable insights into its role within multimodal treatment frameworks. Furthermore, integrating neuroimaging and psychophysiological measures may help clarify the mechanisms underlying music-induced analgesia, while identifying biomarkers that predict treatment response and guide personalized interventions. Advancing this field will require a collaborative effort among neuroscientists, clinicians, music therapists, and patients to ensure that interventions are not only evidence-based but also meaningful, scalable, and accessible.

Several limitations must be acknowledged. Heterogeneity in study methodologies, including differences in musical genres, duration of exposure, and outcome measures, complicates the synthesis of findings and limits generalizability. Many studies rely heavily on self-reported pain ratings, which, while valuable, are subject to bias and may not reflect underlying physiological changes. Moreover, the lack of standardized protocols makes it difficult to compare interventions across populations and settings. Another important limitation is the underrepresentation of diverse patient groups in clinical trials, particularly those from non-Western cultural backgrounds, where musical preferences and meanings may differ significantly. This gap highlights the need for culturally sensitive approaches that account for the social and symbolic dimensions of music in pain perception and coping.

From a clinical perspective, the integration of music listening into pain management protocols offers several advantages but also poses practical challenges. Music is inherently flexible and can be adapted to individual preferences, making it a highly personalized intervention. However, its successful implementation requires careful consideration of patient engagement, therapeutic context, and interdisciplinary collaboration. Clinicians must be equipped not only with knowledge of music's therapeutic potential but also with strategies to assess musical responsiveness and tailor interventions accordingly. In settings where access to specialized music therapists is limited, digital platforms and self-guided listening programs may provide scalable alternatives, though their efficacy and adherence require further evaluation. Importantly, music should not be viewed as a standalone solution or as an inherently easy intervention, but as part of a broader multimodal strategy that includes psychological, physical, and pharmacological components. When integrated thoughtfully, music-based interventions can enhance emotional resilience, reduce reliance on analgesics, and contribute to a more holistic and sustainable model of chronic pain care.
